# Use of wound infusion catheters for delivery of local anesthetic following standing partial ostectomy of thoracolumbar vertebral spinous processes in horses is not associated with increased surgical site infections

**DOI:** 10.3389/fvets.2024.1436308

**Published:** 2024-07-05

**Authors:** Francesca A. Wickstead, Peter I. Milner, David A. Bardell

**Affiliations:** ^1^Department of Small Animal Clinical Sciences, Institute of Infection, Veterinary and Ecological Sciences, University of Liverpool, Neston, United Kingdom; ^2^Department of Equine Clinical Sciences, Institute of Infection, Veterinary and Ecological Sciences, University of Liverpool, Neston, United Kingdom

**Keywords:** wound catheter, local anesthetic, horse, ostectomy, vertebral spinous process

## Abstract

**Background:**

Wound infusion catheters (WICs) have been used in humans and some veterinary species for post-operative local anesthetic administration following a variety of surgical procedures, aiming to reduce post-operative analgesia requirements and improve patient comfort. Benefit in reduction in pain, post-operative analgesia requirements and length of hospital stay are well documented in humans, but use of WICs may not have been widely adopted in veterinary species due to the concern of increased complications, such as dehiscence or surgical site infection (SSI), creating a barrier to their use. This study aimed to evaluate the use of WICs in horses undergoing standing partial ostectomy surgeries, document complications and investigate if the incidence of SSI was equivalent between those horses that did and did not have a WIC.

**Methods:**

Clinical records were searched between January 2010–December 2023 for horses undergoing standing partial ostectomy surgery of thoracolumbar vertebral spinous processes at one institution. Population variables (age, breed, bodyweight), placement of a WIC or not, post-operative complications, analgesia protocols and surgical time were recorded. Horses received up to 0.1 mg kg^−1^ bupivacaine (0.5 mg mL^−1^) every 6–8 h via the WIC where one was placed. To compare SSI complication incidence between using or not using a WIC, a proportional independent equivalence test was used.

**Results:**

There were 64 horses included in the final analysis with a WIC placed in 29/64 horses (45.3%) and 35/64 (54.7%) having no WIC placed at surgery. Incidence of short-term SSI was 11.4% (no WIC used) and 13.8% (WIC used), respectively. The difference in proportion of SSI between the presence or absence of a WIC was not significant [−0.024 (90% CI −0.181; 0.133); *p* = 0.94].

**Conclusion:**

The incidence of SSIs was equivalent between groups whether a WIC was used or not. WICs should be considered as part of a multi-modal analgesic approach in the post-operative period. Further research into local anesthetic dosing and its impact on rescue analgesia requirements and pain-scores is warranted.

## Introduction

Managing post-operative pain in the horse can be a challenge due to the ability to accurately recognize changes in pain status following surgical intervention (1). Often post-operative management involves the use of systemic administration of analgesics which in themselves may result in unwanted side-effects. Additionally, horses affected by conditions resulting in chronic pain have modulated pain pathways, potentially limiting the efficacy of systemically administered drugs (2).

Partial ostectomy is a recognized surgical technique for management of impingement of the vertebral spinous processes (“kissing spines”) in the thoracolumbar region. Originally performed under general anesthesia (3, 4) this procedure is now commonly undertaken with regional anesthesia in the standing sedated horse (5, 6). Due to the invasive nature of the procedure and the removal of sections of bone from multiple processes, post-operative complications can include acute post-operative discomfort, swelling and surgical site infection. Providing adequate analgesia in these patients, particularly in the immediate post-operative period is essential but difficult to achieve.

Wound infusion catheters (WICs) have been used routinely for post-operative local anesthetic administration in humans, with significant reductions in opioid requirement noted following surgery (7–9). Use of WICs for provision of post-operative analgesia in dogs and cats following limb amputation, total ear canal ablation and injection site sarcoma resection has been described, but similar benefits are not reported (10–12). The use of WICs has not been widely adopted for post-operative pain management in equine surgery, potentially due to concerns over increased wound complications such as dehiscence, infection and seroma formation. The objective of this study was to evaluate the use of WICs in a cohort of horses undergoing standing partial ostectomy of thoracolumbar vertebral spinous processes, and document post-operative complications compared to horses undergoing the same procedure, but without a WIC being used. Our hypothesis was that there is equivalence in incidence of short-term surgical site infection (SSIs) in horses undergoing partial ostectomy of the vertebral spinous processes whether a WIC was used or not.

## Materials and methods

### Data collection

Institutional ethical approval (VREC 1423 date: 10.31.23) was obtained for this study. Clinical records for horses admitted to the Leahurst Equine Hospital, University of Liverpool, UK between January 2010 and December 2023 for surgical management of impingement of thoracolumbar spinous processes by partial ostectomy under standing sedation were inspected. Explanatory variables collected included age (years), sex, breed, bodyweight (kg), surgeon, surgical time (minutes), number of spinous processes operated on, post-operative analgesia used, presence of surgical site infection (yes/no), presence of moderate/marked swelling (yes/no), time from surgery to hospital discharge (days). Surgical site infection (SSI) was defined as purulent discharge from the incision, positive bacterial culture from the incision and/or evidence of wound breakdown (or deliberate opening of the surgical incision to allow drainage) (13), prior to hospital discharge. Cases were grouped into whether a WIC was used or not.

### Surgical method and wound infusion catheter protocol

All horses were administered acepromazine [0.03 mg kg^−1^ intramuscular (IM), Tranquinervin, Dechra] 45 min prior to surgical preparation. Flunixin meglumine [1.1 mg kg^−1^ intravenous (IV), Finadyne, MSD] and either procaine penicillin (20 mg kg^−1^ IM, Depocillin, MSD), gentamicin sulfate (6.6 mg kg^−1^ IV, Genta-Equine, Dechra), or oxytetracycline (7.5 mg kg^−1^ IV, Engemycin, MSD) were administered 30 min prior to the start of surgery. A cannula was placed in the left jugular vein and horses were administered xylazine (0.5 mg kg^−1^ IV, Virbaxyl, Virbac), romifidine (0.05 mg kg^−1^ IV, Sedivet, Boehringer Ingelheim) or detomidine (0.008 mg kg^−1^ IV, Equimidine, Zoetis) as an intravenous bolus and continued as an intravenous infusion, alongside morphine sulfate (0.2 mg kg^−1^ IV, Morphine sulfate, Martindale Pharma) during the procedure. Mepivicaine hydrochloride [Intra-epicaine (2%), Dechra] was infiltrated around the surgical site (4 mL cm^−1^; up to 8 mg kg^−1^) 10 min prior to the start of surgery. Surgery consisted of a midline incision through the skin, subcutis and supraspinous ligament to access the affected spinous processes. Partial ostectomy of relevant processes was performed using a combination of oscillating saw and rongeurs. Closure of the supraspinous ligament, subcuticular and skin layers was then performed. Post-operatively all horses received 4.4 mg kg^−1^ phenylbutazone IV twice daily for 24 h, reducing to 2.2 mg kg^−1^ twice daily PO. Systemic antimicrobials were continued for 3–5 days.

**Figure 1:**
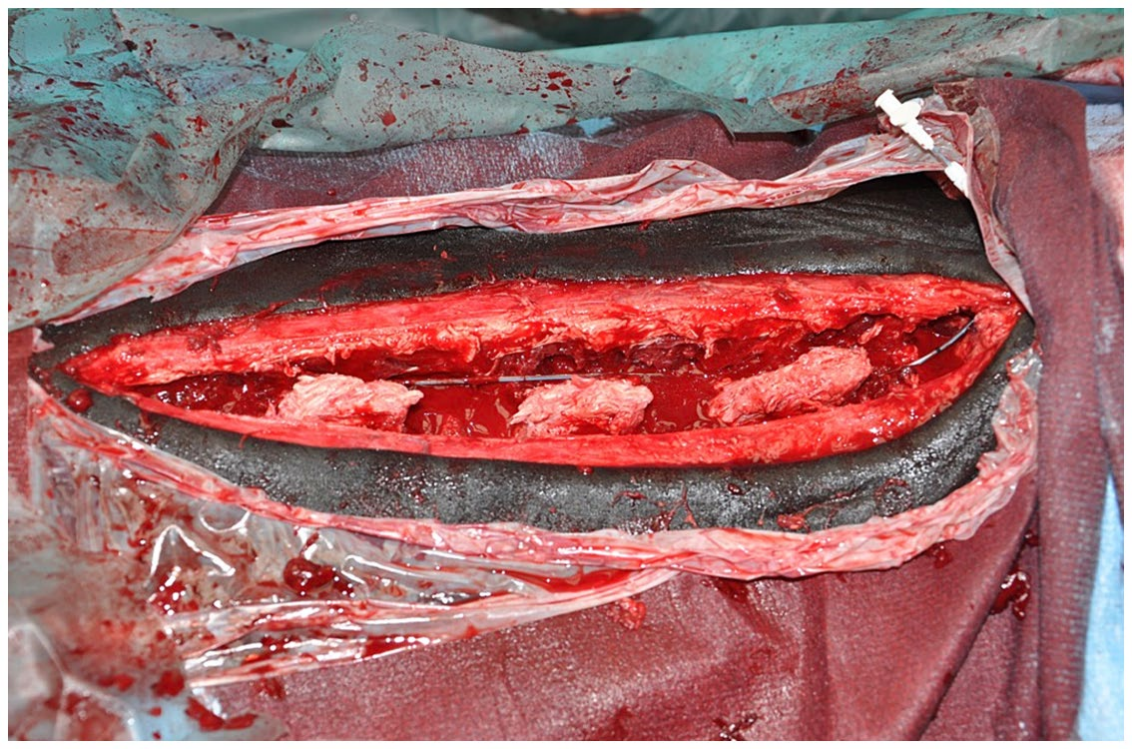
Wound infusion catheter *in situ* at end of surgery, prior to closure (cranial is to the right).

**Figure 2:**
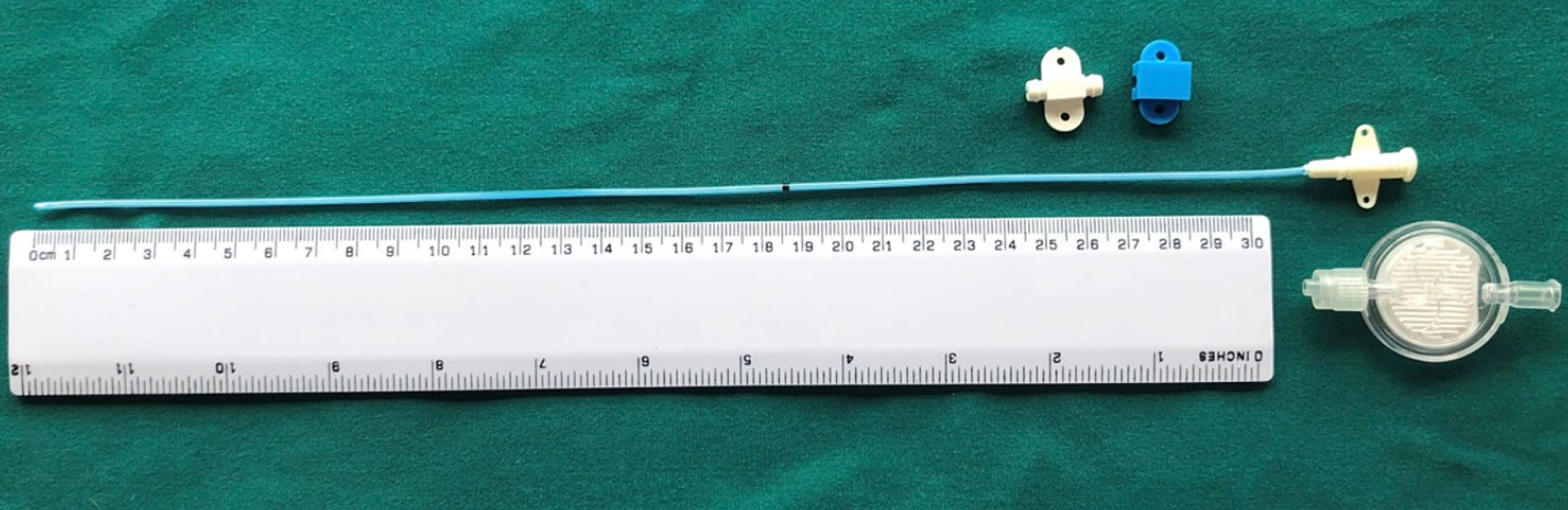
Veterinary MILA wound diffusion catheter and filter.

**Figure 3:**
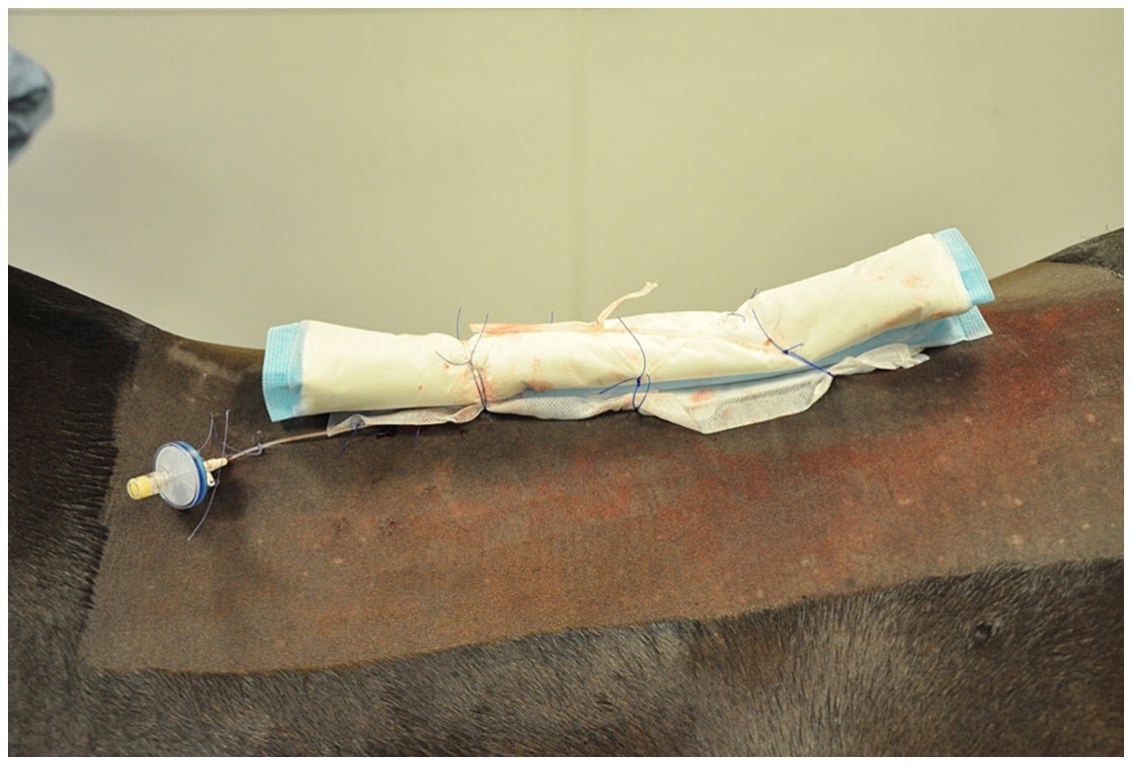
Wound infusion catheter and filter in place at end of procedure (cranial is to the left).

For horses where a WIC was used, a 16 gauge diffusion catheter (Mila, Kentucky, United States) was placed along the length of the resected spinous processes and exited through a separate skin incision approximately 3 cm to the left of the cranial extent of the surgical incision (Figure 1). Catheters had either a 6, 7.5 or 9 inch dispersion length according to length of surgical field (Figure 2) and a 0.2 μm filter (Smiths Medical ASD Inc., Minnesota, United States) was secured to the catheter and a cruciate suture pattern used to anchor the filter in place (Figure 3). Bupivicaine [Marcain (0.5%), AstraZeneca] was administered via the WIC at a volume of 0.3 mL cm^−1^ of surgical wound (equivalent of up to 0.1 mg kg^−1^), at the end of the procedure and then every 6 h for the first 24 h, followed by every 8 h for the next 24 h, under sterile conditions. The surgical site was covered with a non-absorbent adhesive dressing (Primapore, Smith & Nephew) which was protected with an oversewn stent bandage. Dressings were changed every 12 h and assessed for discharge or dehiscence. The WIC filter was covered with an adhesive dressing (Opsite, Smith & Nephew) and sterile bung. The WIC was removed after 48 h in all cases. Inclusion of a WIC was non-randomized and based on surgeon preference.

### Statistical analysis

Data are presented as mean (95% CI) or median (IQR). Statistical analysis was performed using SPSS statistical software (version 29 for Windows, IBM, Chicago, IL, United States). Distribution of data was assessed using visual inspection of histograms, Q-Q plots and Shapiro–Wilk test for normality and Levene’s test for equality of variance. Unpaired student’s *t*-test or Mann Whitney *U* test were used for univariable analysis of continuous data. Fisher’s exact test was used to compare categorical data between groups. An adjusted *p*-value of 0.05/12 = 0.004 was used after Bonferroni correction. To compare complication incidence of SSI between using/not using a WIC, independent proportional equivalence testing was used (14). Results of the equivalence test are presented as proportional difference with 90% CI based on two one-sided testing with upper and lower limits of equivalence set as +/− 0.20. Post-hoc sample size calculation indicated 28 cases per group were required to achieve a significance of 0.05 and power of 0.8 (15).

## Results

### Descriptive analysis

There were 64 horses included in the final analysis with a WIC placed in 29/64 horses (45.3%) and 35/64 (54.7%) having no WIC placed at the end of surgery. Mean age of horse included in the study was 9.3 years (95% CI 8.3–10.2) with 38 geldings and 26 mares. Most breeds (34/64) were Thoroughbred or Thoroughbred cross (TB/TBX). There were 11 different surgeons involved with two surgeons performing most surgeries (42 and 32%, respectively). Days to discharge had a median of 5 days (IQR 4–6). The number of vertebral spinous processes operated on were a median of 4 (IQR 3–5), consisting of T13-L3.

There was no significant difference in age, sex, breed, bodyweight, number of spinous processes operated on, surgical time or days to discharge between groups with or without a WIC. There was a significant difference in surgeons using a WIC with one surgeon responsible for using WICs in 24/29 cases (*p* < 0.001). Table 1 summarizes the continuous clinical data for groups with and without a WIC.

**Table 1:** Univariable analysis of continuous explanatory variables for horses undergoing partial spinous process ostectomy surgery with and without a wound infusion catheter (WIC).

Variable	Non-WIC	WIC	*p*-value
Surgical time (minutes)	111 (96.5–126.2)	107 (94–119.9)	0.66
Days to discharge (days)	5.2 (4.5–6)*	5.7 (4.9–6.4)*	0.24†
Number of SPO	3.5 (3–4)*	4.4 (3.7–5.1)*	0.05†
Bodyweight (kg)	539.0 (514.7–563.3)*	573.4 (541.9–604.8)*	0.07†
Age (years)	9.8 (8.4–11.1)	8.8 (7.4–10.3)	0.33

Independent *t*-tests were used for normally distributed and Mann Whitney *U* tests † for non-normally distributed data. A Bonferroni corrected *p* < 0.004 indicates significance. Data presented as mean (confidence interval 95%) or median (IQR)*. SPO, spinous process ostectomy.

### Surgical site infection

All horses with WICs tolerated placement and no interference with the catheter post-operatively was reported. SSI was reported in 4/29 (13.8%) horses with a WIC and in 4/35 (11.4%) horses without a WIC. When present, all instances of SSI responded successfully to antimicrobial therapy. The presence, or absence of a WIC did not alter the proportion of horses with SSI [−0.024 (90%CI −0.181; 0.133); *p* = 0.94]. There were no reports of moderate to marked swelling of the surgical site in horses without SSI.

### Other post-operative complications

Signs of colic were reported in 2/29 (6.9%) horses with a WIC and in 4/35 (11.4%) horses without (no significant difference between groups, *p* = 0.68). In most cases, signs were described as mild and responded to medical management within 24 h. One horse in the non-WIC group developed persistent and severe colic signs and underwent exploratory laparotomy 48 h post-surgery, at which time cecal rupture was diagnosed and the horse euthanized. The horse was euthanized, for clinical reasons not related to this study, with IV cinchocaine hydrochloride quinalbarbitone sodium (Somulose, Dechra). All other data from this patient was included for analysis.

Other complications were reported in 2/29 (6.9%) horses with a WIC. One horse developed a head tilt and circling 24 h after WIC removal which resolved spontaneously, and one horse had an elevated heart rate after WIC removal, which responded to phenylbutazone administration and was ascribed to pain. In the non-WIC group, 2/35 (5.7%) horses showed other complications. One horse had an episode of esophageal obstruction which resolved spontaneously, and one horse developed pyrexia for 24 h which resolved without any change in post-operative management.

## Discussion

This study found an equivalent incidence of surgical site infections (SSIs) following spinous process ostectomy surgery in horses irrespective of whether a wound infusion catheter (WIC) was placed for administration of bupivacaine for post-operative pain management or not.

Concerns of increased seroma formation, wound drainage, delayed wound healing and increased wound infection rates in relation to the use of WICs have been proposed, but have not been proven in human or veterinary literature (16). Where complications have been reported, they have been described as minor, including seroma formation and patient disconnection from infusion delivery systems, without increased infection rates (10, 12). Abelson et al. (10) reported a 5.5% incidence of SSI with WICs compared to 15% in historic case controls without WICs. No incidents of catheter interference by horses were identified in this study.

Incidence of SSI was 12.5% overall (13.8% in horses with a WIC and 11.4% in those without), in line with previous reports for this surgery (3.5–20%) (4, 15) and similar to studies utilizing WICs in other veterinary species (10, 12, 17). SSIs can lead to wound breakdown, delayed healing, increased patient discomfort, cosmetic and financial implications. All cases in this study received pre-operative antimicrobial treatment which was continued for at least 3 days post-operatively (procaine penicillin, gentamicin sulphate, oxytetracycline). All SSIs resolved with dressing management and antimicrobial therapy based on culture and sensitivity results.

Post-operative colic signs were mild in all cases except one in the non-WIC group, in which cecal rupture occurred resulting in euthanasia of the horse. Colic signs are multi-factorial and include change in housing and management, sedation, pain, stress, and other underlying co-morbidities. Horses that displayed signs of colic had surgery times close to the mean, without excessive use of opioids or alpha-2 agonists. Interestingly, another horse, also in the non-WIC group, developed esophageal obstruction, which resolved spontaneously, making gastrointestinal dysfunction the most frequently reported non-SSI post-operative complication in this study.

Due to the limited duration of action of commonly used local anesthetic agents, repeat administration or continuous infusion is required. Bupivacaine administration in those horses where a WIC was placed, was based on volume per unit length of surgical incision (0.3 mL cm^−1^, 0.5% bupivacaine) delivered at predefined time points. This was empirically derived and based on surgical closure of dead space requiring minimal volume sufficient to permeate the surgical site and remain below accepted toxic doses, whilst achieving a useful effect. Other authors have reported using lidocaine at 1.2–3 mg kg^−1^ h^−1^ (10, 12) and bupivacaine 1.5 mg kg^−1^ (10, 16) and 0.13–0.21 mg kg^−1^ h^−1^ (17). Effective blockade of the palmar nerves in the equine distal limb has been reported following continuous perineural infusion of 0.5% bupivacaine at 4 mL hr^−1^ (18) and perineural bolus deposition of 3–4 mL of 1.25–2.5 mg mL^−1^ solutions (19). Described in these terms, our dosing regime equates to <0.1 mg kg^−1^ every 6–8 h, so may have been excessively conservative. We utilized commercially available, veterinary specific WICs with variable dispersion lengths (6, 7.5 and 9 inches). Dispersion length was chosen to match incision length as closely as possible, to maximize drug distribution throughout the surgical site, but uniformity of distribution is unknown.

Local anesthetic toxicity can manifest as neurotoxicity, and one horse in the WIC group exhibited neurological signs 24 h after WIC removal. The bupivacaine dosing regime, coupled with the time frame between administration and onset of clinical signs, make it unlikely this was due to systemic absorption of the local anesthetic. The total dose of bupivacaine received was 0.28 mg kg^−1^ over 48 h. Toxic doses of bupivacaine previously reported vary widely between species and route of administration. In dogs 10 mg kg^−1^ IV resulted in cardiotoxicity (20), whilst in humans cardiovascular collapse requiring resuscitation is reported following 2–3 mg kg^−1^ administered for regional anaesthesia (21). The doses in this study were much lower (up to 0.1 mg kg^−1^), clinical signs spontaneously resolved rapidly, and the cause remains unknown. Radlinsky et al. (17), using a dosing regime of <0.21 mg kg^−1^ h^−1^ (up to 4.3 mg kg^−1^ day^−1^) were unable to detect bupivacaine in the plasma of dogs and reported no signs of drug toxicity. Abelson et al. (10) reported one dog with signs of toxicity five hours after starting lidocaine infusion at 1.78 mg kg^−1^ h^−1^, which resolved after the infusion was discontinued.

Local anesthetics bupivacaine, lidocaine and mepivacaine have been demonstrated to have antibacterial properties. Testing with common equine pathogens inhibited growth up to 93% *in vitro*, with the majority of concentrations achieving bactericidal action, lidocaine being the most potent, followed by bupivacaine, then mepivacaine. Bupivacaine 2.5 mg mL^−1^ (half of the concentration used in this study) inhibited 93% of bacterial isolates (22). Bacterial culture of WIC tips performed by Radlinksy et al. (17) produced positive results in only one dog out of ten. None of the dogs in that report exhibited clinical signs of infection and none required treatment. Perineural catheters placed in the distal limb of horses resulted in swelling which the authors attributed to venous dilation caused by the local anesthetic agent reducing lymphatic drainage and venous return (18, 19). This effect was less apparent with bupivacaine than lidocaine, resolved with discontinuation of infusion and had no further implications (18, 19).

Due to the retrospective nature of this study, we were unable to investigate the impact of WIC use on analgesia requirements, as horses were not routinely pain scored post-operatively and analgesia protocols were largely standardized regardless of WIC use. One horse in the WIC group exhibited an episode of tachycardia which responded to bringing the planned administration of phenylbutazone forward, so was ascribed to a pain response, but this occurred 2 days after WIC removal.

Local anesthetic infiltration of surgical wounds via WICs is reported to improve comfort and reduce opioid requirements in humans undergoing limb surgery, breast surgery, laparoscopy/laparotomy of the abdominopelvic area and cardiothoracic surgery. Greatest benefit is derived in treatment areas of subcutaneous, cutaneous and connective tissue structures, whilst in complex structures such as articulations and areas with multiple innervations, response is less dramatic, but still results in reduced opioid consumption (23–25). In veterinary species, it is unknown if opioid requirements and rescue analgesia differ post-operatively with WIC placement (12, 17). This could be due to several reasons; limited variety of surgical procedures studied; other analgesics used concurrently with the local anesthetic agent, inadequate dosing of local anesthetic, varied anesthetic and analgesic protocols and varied compliance with pain scoring. Additionally, wound inflammation and infection may interfere with local anesthetic efficacy. Some authors have described recording lower pain scores in dogs and horses with WICs, but these differences did not achieve statistical significance and were not sufficient to elicit changes in opioid or rescue analgesia requirements (12, 26).

We were not able to demonstrate an advantage in terms of reduced duration of hospitalization in horses with WICs. The horse with the longest period of hospitalization was in the non-WIC group, but discharge from the hospital was delayed by owner availability, not surgery-related factors. In humans, local anesthetic administration in the post-operative period can reduce length of hospital stay, but this is yet to be demonstrated in veterinary species (27).

This study was subject to limitations common to retrospective analyses, namely similar, but non-standardized sedation and analgesia protocols, inconsistent and largely absent pain scoring, limited and inconsistent clinical notes and lack of long-term follow-up. The use of WICs in horses for post-operative pain management following subtotal spinous process ostectomy surgeries warrants further study. Future work should seek to establish safe and effective local anesthetic administration regimes and determine any potential for reduction in systemic analgesic administration by using rigorous assessment of comfort levels and pain-associated behaviors. If patient comfort is improved by use of WICs and local anesthetic administration, this may lead to quicker hospital discharge, reduced financial costs and improved welfare.

In conclusion, WICs used post-operatively in spinous process ostectomy surgeries did not have a negative impact of incidence of SSI prior to hospital discharge and are easily managed in a hospital setting.

## Data availability statement

The original contributions presented in the study are included in the article/supplementary material, further inquiries can be directed to the corresponding author.

## Ethics statement

The animal studies were approved by Veterinary Research Ethics Committee—University of Liverpool. The studies were conducted in accordance with the local legislation and institutional requirements. Written informed consent was obtained from the owners for the participation of their animals in this study.

## Author contributions

FW: Data curation, Investigation, Writing – original draft, Writing – review & editing, Formal analysis. PM: Conceptualization, Supervision, Writing – original draft, Writing – review & editing, Formal analysis. DB: Conceptualization, Supervision, Writing – original draft, Writing – review & editing.
